# Treatment of Psoriasis

**Published:** 1873-08

**Authors:** 


TREATMENT OF PSORIASIS.—Psoriasis is not in itself a dangerous disease, says M. A. de Montméja, but it is obstinate, and those who have been once affected by it are very liable to relapses. It is often hereditary, manifesting itself only when the adult period is reached, after which it may be either intermittent or inveterate. In the present state of our therapeutical knowledge we must not imagine that we can effect a radical cure of psoriasis; we may clear the skin, or hasten the evolution of an attack, but it is impossible to prevent relapses. The treatment is divisible into local and general means. The general treatment consists in the administration of mild and frequently repeated aperients and of arsenical and sulphuretted preparations, as well as of those containing cantharides. M. Hardy prefers small doses of the arseniate of soda to the other preparations of arsenic. M. de Montméja has obtained considerable success from the employment of two drops of tincture of cantharides in a glass of eau sucrée, the dose being increased up to thirty drops per diem. Its use, however, requires extreme care and vigilance. Copaiva is sometimes very useful when given internally.

